# Effect of steam explosion on nutritional components, physicochemical and rheological properties of brown rice powder

**DOI:** 10.3389/fnut.2022.954654

**Published:** 2022-08-22

**Authors:** Feng Kong, Qinghua Zeng, Yue Li, Xue Di, Yishuai Ding, Xingfeng Guo

**Affiliations:** Agricultural Science and Engineering School, Liaocheng University, Liaocheng, China

**Keywords:** steam explosion, brown rice, starch digestibility, rheological property, structural characteristic

## Abstract

Brown rice powder is underutilized mainly due to its lower starch digestibility and poor processing performance. The present study investigated the potential of steam explosion on the improvement of nutritional and physicochemical characteristic in brown rice powder and rheological property of paste. Compared with native brown rice powder, steam explosion at 0.5 MPa for 7 min increased the water-extractable arabinoxylans (5.77%), reducing sugar content (21.04%), and iodine blue value (30.38%), which indicated steam explosion that destroyed the intact cells of brown rice. Later the crystalline structure of brown rice powder was destroyed into an amorphous structure by steam explosion. Steam explosion enhanced the degree of gelatinization (4.76~351.85%) and solvent retention capacity (SRC) of brown rice powder, compared with native sample. The effect on the intact cells and starch structure of brown rice caused the starch digestibility enhancement remarkable. Viscoelastic profiles confirmed that steam explosion weakened the paste strength and elasticity corresponded with hardness and cohesiveness by increasing the loss factor (tanδ). This work provided important information for brown rice powder modified by steam explosion (0.5 MPa, 7 min) with good nutritional property (nutrients and digestibility) and processability (SRC, textural, and rheological property). Steam exploded brown rice powder (0.5 MPa, 7 min) could serve as a potential ingredient widely used in food products.

## Introduction

Brown rice has been categorized as one of the potent functional foods because of its high bioactive compounds content and biological activity (1). As an important ingredient of whole grain food, long-term consumption of brown rice was good for health, which can effectively reduce the risk of cardiovascular and cerebrovascular diseases, diabetes, and other related chronic diseases (2). However, brown rice was rarely consumed as it is covered by the rice bran layer, which caused its poor cooking and eating quality (3, 4). Like other cereal flours, brown rice powder faced some problems, such as how to improve starch digestibility and rheological property. The lower starch digestibility/bioaccessibility associated with intact cells or tissues (5). Rice bran prevented the rapid infiltration of water, resulted in long time cooking (4) and hindered the digestion of macro-nutrients such as starches, and the brown rice paste emerged the high viscosity; so it was difficult to be accepted by consumers (6). It is necessary to find a method which is efficient to improve the digestibility and rheological property of brown rice powder.

Recently, researchers have shown an increased interest in the steam explosion applications. Steam explosion is a novel hydrothermal processing technology in food industry with high efficiency and low energy consumption, which was often employed in lignocellulosic or high-protein materials (7–9). Steam explosion pretreated material by high-pressure saturated steam for a set of period of time and then released the pressure instantaneously; it could thermally mechanically modify the feedstock. Steam explosion broke the cell wall structure, led to the increase of soluble dietary fiber content, and reduce lipase activity and phytic acids of wheat bran (9–11). And steam explosion hydrolyzed the glycosidic bonds and decreased the molecular weight in potato starch (12). Therefore, steam explosion treatment maybe an effective method to improve the quality of brown rice powder in food industry, steam exploded brown rice may be incorporated into various food products including beverages and fermented condiments. However, the effect of steam explosion on the physicochemical property of brown rice powder has never been studied.

The aim of this work was to investigate the effect of steam explosion on improvement of the digestibility and rheological property of brown rice powder. The effects of steam explosion on the structural characteristic (scanning electronic microscopy and X-ray diffraction), nutritional characteristic (chemical compounds and *in vitro* starch digestibility), and processibility (solvent retention capacity (SRC) and textural and rheological properties) of brown rice powder were evaluated. These results upon evaluation of nutritional, physicochemical, and rheological characterization in the investigated brown rice powders will have the guiding meaning for the application of steam explosion as an innovative modifying technology in the cereal processing industry and help to identify a new source of flour in food applications for the development of whole grain products from brown rice powder.

## Materials and methods

### Sample preparation

Steam explosion was performed using a self-designed batch vessel that consisted of a reaction chamber (WY19, Big Soldier Food Machinery, Henan, China) and a steam generator (WY19, Big Soldier Food Machinery, Henan, China) (13). 500 g of brown rice (Inner Mongolia Deluke Agricultural Development Co., Ltd., Inner Mongolia, China) was loaded into the reactor chamber and treated at 0.3~0.7 MPa for 3~7 min; the holding temperature was ranged from 133 to 165°C, the time for increasing pressure to set pressure was kept under 10 s. The reaction system was then terminated with a sudden explosion by opening outlet valve; the brown rice was allowed to dry at 60°C for 12 h. The 100 g dried brown rice was ground for 2 min in ZT-150 high-speed grinder (Yongkang Zhanfan Industry and Trade Co., Ltd., Zhejiang, China).

### Morphological analysis of brown rice powder

The scanning electronic microscopy (SEM) (Thermo Scientific, Helios G4 CX) was used to obtain SEM images of brown rice powder at a voltage of 1.00 kV.

### X-ray diffraction analysis of brown rice powder

The structures of brown rice samples were measured using a diffractometer (Rigaku, SmartLab 9 kW). The samples were scanned from 5 to 80° with a scanning speed of 0.02°/s.

### Color measurements of brown rice powder

The color profiles of native and steam exploded brown rice powders were measured with a chromameter (Minolta CR-10, Japan). L^*^ means lightness of the powder, a^*^ indicates green or red-purple color and b^*^ indicates yellow or blue color (14). Chroma (15), hue (16), total color difference (ΔE) (15), and browning index (17) were calculated using L^*^, a^*^, and b^*^ values.

### Chemical compounds of brown rice powder

Protein content of brown rice powder was analyzed using AACC method (46-08). Total arabinoxylans and water-extractable arabinoxylans contents were determined using the Hashimoto's method (18). The reducing sugar content was estimated with the 3'5-dinitrosalicylic acid method (19) with modifications. Briefly, brown rice powders (1 g) were dispersed in distilled water by shaking in a 50°C water bath for 2 h. The dispersion was filtrated and made to 50 ml with deionized water. The extract (1 mL) and DNS solution (2 mL) were mixed and heated in a boiling water bath for 5 min. The absorbance of the solution was measured at 540 nm. Starch content of brown rice powder was measured by the Goñi's method (20) with modifications. Briefly, brown rice powders (0.1 g) were mixed with 2 mol/L of KOH solution (6 mL) and shaken at 25°C for 30 min. Then, 98% of acetum (1 mL), 0.4 mol/L of sodium acetate buffer solution (pH = 4.75, 3 mL), and amyloglucosidase were added (from Aspergillus niger, 1 × 10^5^ U/mL, 200 μL) and incubated at 60°C for 45 min. Iodine blue value was estimated using protocol established by the method (21); brown rice powders (0.25 g) were mixed with 50 mL of heated water (65.5°C) and incubated in the water bath at 65.5°C for 5 min. Filtrate (5 mL) was mixed with 0.02 mol/L of iodine standard solution (1 mL) and made to 50 mL, then the absorbance was noted at 620 nm. Gelatinization degree of brown rice powders was determined by Birch and Priestley method (22) with modifications. Briefly, brown rice powders (0.1 g) were dispersed in 49 mL deionized water, 10 mol/L of KOH solution (1 mL) was added and centrifuged at 5,000 rpm for 10 min. Supernatant (1.0 mL) was added with 0.5 mol/L of HCl solution (0.4 mL) and made to 10 mL with deionized water and added 0.1 mL of iodine solution. The absorbance was noted at 600 nm and gelatinization degree was expressed as the absorbance value ratio of determined sample to control sample.

### *In vitro* starch digestibility of brown rice powder

Starch digestibility was determined as the previous report (20, 23–26) with modifications. α-Amylase (6 g, 35 U/mg) (Shanghai Macklin Biochemical Co., Ltd., Shanghai, China) and 40 mL of deionized water were mixed followed by stirring for 10 min. Then, the mixed solution was centrifuged at 4,000 rpm for 15 min. Finally, 30 mL of the supernatant was mixed with 2 mL of amyloglucosidase (from Aspergillus niger, 1 × 10^5^ U/mL) (Shanghai Macklin Biochemical Co., Ltd., Shanghai, China) and 3 mL of deionized water to prepare digestive enzyme solution. Brown rice powders (500 mg) was mixed with 14 mL of deionized water and boiled in a water bath for 5 min. Then, 1 mL of digestive enzyme solution was added, shaken, and incubated at 37°C. Sample solutions of 0.2 mL were taken out after 20, 60, 120, 180, and 240 min and added 80% ethanol solution (5 mL) immediately.

### Solvent retention capacity of brown rice powder

The SRC tests, included lactic acid (LA), sodium carbonate (SC), sucrose (Suc), and water (W), were measured according to the AACC International Approved Method 56-11.02.

### Textural property of brown rice powder

The pastes were prepared in the 100-mL centrifugal tube with 6 g powders and 60 mL deionized water; the suspensions were shaken in 95°C water for 30 min and stored at 4°C. The textural properties of pastes were tested by a CT3 Texture Analyzer (Brookfield Engineering Labs, Inc., USA) with cylinder probe of TA4/1000, and the test speed was set at 0.5 mm/s and the degree of deformation of gel was 30%.

### Dynamic rheological property of brown rice powder

The dynamic rheological property of all samples was investigated by an HR-1 Rheometer (TA Instruments, USA) based on a method (27, 28). 6 g powders and 60 mL deionized water were mixed and shaken in 95°C water for 30 min and stored at 4°C. After pasting and cooling, partial of each sample was transferred directly on the parallel plate (40 mm diameter and peltier plate steel). The sweep procedure was run from 0.1~1,000 rad/s at 25°C with strain amplitude of 2%.

### Statistical analysis

Three replicate tests were carried out and the average values were reported. Experimental data were processed by one-way analysis of variance using IBM SPSS Statistics 20 (IBM, NY, USA) with Duncan's multiple range test (*p* < 0.05). The results were reported as mean ± standard deviation.

## Results and discussion

### Microstructural properties of brown rice powder

**Figure 1:**
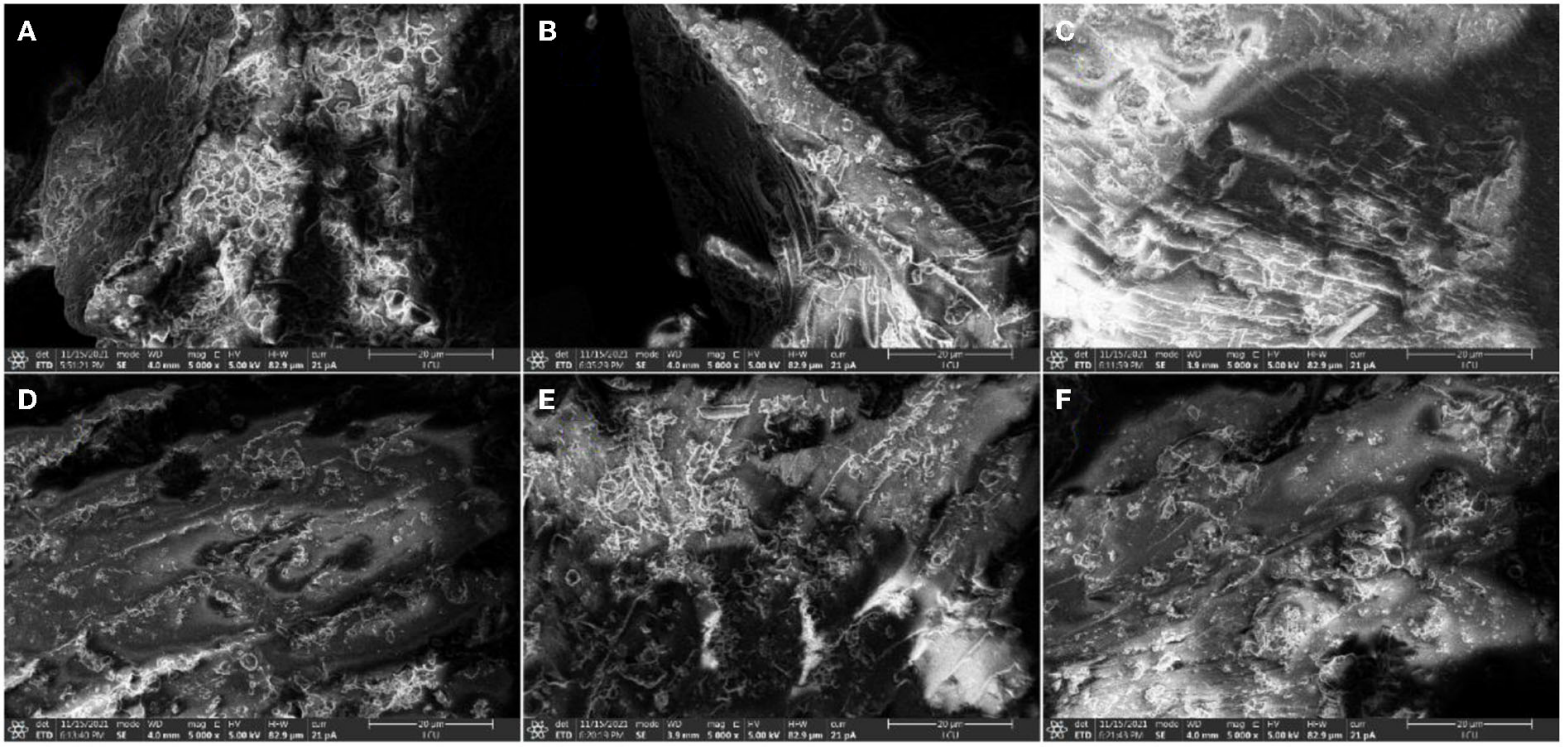
SEM images of brown rice powders. NBR: native brown rice powder, SBR: steam exploded brown rice powder, **(A)** NBR, **(B)** SBR at 0.3 MPa for 5 min, **(C)** SBR at 0.5 MPa for 3 min, **(D)** SBR at 0.5 MPa for 5 min, **(E)** SBR at 0.5 MPa for 7 min, **(F)** SBR at 0.7 MPa for 5 min.

The morphological changes of brown rice powders are investigated in Figure 1. Steam explosion treatment caused visible changes in the morphology of brown rice powders. The steam exploded brown rice powders showed a less rounded starch form and took a smaller form after exposure to steam than that of native brown rice. Under the double action of high temperature and high pressure of saturated steam during steam explosion process, brown rice starch granules were extensively swollen after absorbing a large amount of water. Then, the swollen granules broken and formed the gel during steam heating and cooling (29). This gelatinized starch gel of steam exploded brown rice could be regarded as a continuous matrix with few residual granules surrounded by free starch molecules, which might appear as the viscoelasticity and shear-thinning behavior (30).

### X-ray diffraction analysis of brown rice powder

**Figure 2:**
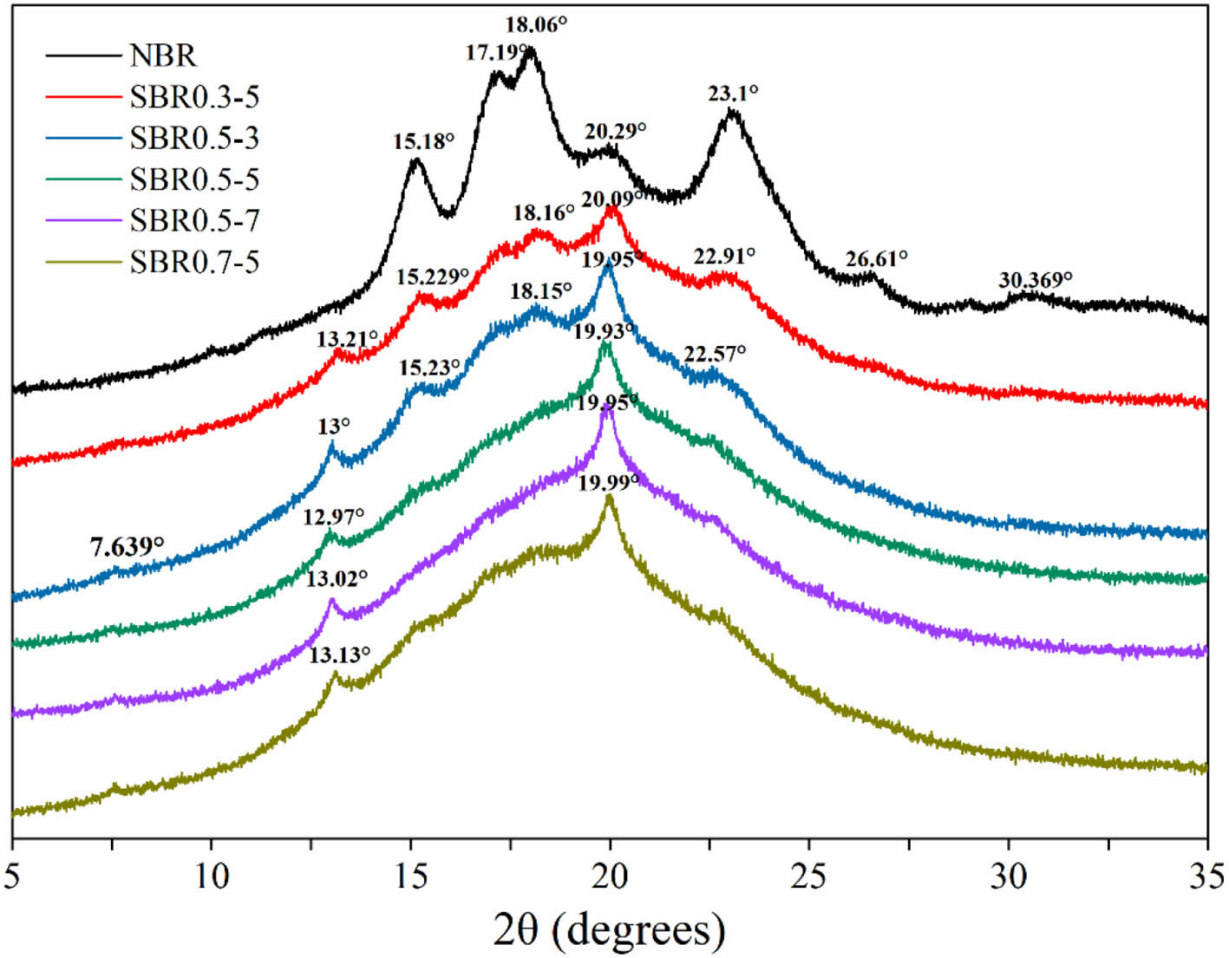
XRD patterns of native and steam exploded brown rice powder. NBR, native brown rice powder; SBR, steam exploded brown rice powder; SBR0.3-5, SBR at 0.3 MPa for 5 min; SBR0.5-3, SBR at 0.5 MPa for 3 min; SBR0.5-5, SBR at 0.5 MPa for 5 min; SBR0.5-7, SBR at 0.5 MPa for 7 min; and SBR0.7-5, SBR at 0.7 MPa for 5 min.

The characteristics of diffraction peak in X-ray diffraction showed the crystal structure and amorphous region; it could be used to judge the crystallization characteristics of brown rice starch granules (31). The crystalline characteristics of the native and steam exploded brown rice powders were studied by XRD (Figure 2). The native brown rice powder exhibited clear diffraction peaks characteristics at the diffraction angle (2θ) of 15.18°, 17.19°, 18.06°, and 23.1°, included double-diffraction peaks at 17.19° and 18.06° and weak small peaks near 20.29°, which indicated the native powder belonged to A-type crystalline pattern (32). After steam explosion treatment, the new peaks at 7°, 13°, and 19.9° indicated that the pattern of steam exploded brown rice powder was V-type crystals (33), which could be attributed to amylose-lipid complex formation during steam explosion process (16, 34). The most of the starch has been gelatinized, and the crystalline structure of brown rice powder was destroyed into an amorphous structure by steam explosion, based on the disappearance of the diffraction peaks (31). A similar observation was made by Li et al. (12); they suggested that the degree of polymerization of molecular chains in sweet potato starch was degraded in steam explosion treatment process (12). The steam explosion at dramatic conditions might cause more severe damage of crystalline structure. The amorphous structure of starch might has a stronger hydrability; steam exploded brown rice powder might have a higher water absorption capacity.

### Color profiles of brown rice powder

The color profiles of L, a^*^, and b^*^ values of brown rice powders are shown in Table 1. L^*^, a^*^, and b^*^values denoted lightness to darkness, redness to greenness, and yellowness to blueness, respectively (35). The steam exploded brown rice samples had a lower L^*^ values, but exhibited no change in a^*^ values without steam exploded sample (0.5 MPa, 7 min) as compared to native powder (L^*^ = 54.43, a^*^ = 38.87, b^*^ = 17.90). Color measurements indicated that steam explosion treatment of brown rice yielded a darker color (lower L^*^ values). A similar result was obtained by Zhao et al. (36), whereas steam exploded wheat bran exhibited a significantly darker color than raw wheat bran. The undesired Maillard browning reaction, caramelization, and oxidation products formation could be responsible for the decrease of lightness in steam exploded powders compared to native powder (11, 28). The yellowness of the steam exploded powders, ranged from 17.03 ± 0.15 to 19.27 ± 0.15, was significantly different from native powder, except for SBR0.5-3 and 0.5-5. The greenness of the steam exploded powders (0.5 MPa, 7 min) was significantly decreased compared with that of native powder. The total color difference (ΔE) of steam exploded brown rice powders varied from 3.51 ± 0.18 to 5.31 ± 0.44, which was typically used to evaluate the degree of the total differences between steam exploded and native brown rice powders. The chroma values of steam exploded powders indicated the purity or saturation, showed no significant variation compared to native powder (15). No change was found in chroma between native and steam exploded brown rice powders (*p* > 0.05), which indicated stability of yellow color in brown rice powders. The Hue angle values increased from about 0.43 to 0.47 during steam explosion process at 0.5 MPa for 7 min. It suggested increment from a more green color to an orange-red color of steam exploded brown rice. The Hue values of steam exploded powders were gradually increased from 0.41 to 0.46 with the rising of treated pressure from 0.3 to 0.7 MPa for 5 min. The browning index represented the purity of brown color which was an important parameter in processes where enzymatic and non-enzymatic browning took place (17). Browning index changed between 91.37 and 100.68 in steam explosion process, which were significantly higher than that of native powder (87.64). These results indicated that the steam explosion strongly affected the color quality of brown rice and produced more browning compound(s). Several investigators have reported similar observations (11, 28, 36). Steam explosion caused changes in color of wheat bran from faint yellow to brown and the color change could be advantageous in cookies (37). There was a remarkable negative correlation (*p* < 0.01) between L^*^ and ΔE (r = −0.987), L^*^ and browning index (r = −0.894), a^*^ and b^*^ (r = −0.785), a^*^ and Hue (r = −0.886), a^*^ and browning index (r = −0.732), while b^*^ and Hue (r = 0.975), b^*^ and browning index (r = 0.757), Hue and browning index (r = 0.759), ΔE and browning index (r = 0.899) showed significant positive relationship (*p* < 0.01).

**Table 1:** Effect of steam explosion on the chrominance of brown rice powder.

**Samples**	L*	a*	b*	**ΔE**	**Hue**	**Chroma**	**Browning index**
NBR	54.43 ± 0.81^a^	38.87 ± 0.23^ab^	17.90 ± 0.10^c^	0.00 ± 0.00^e^	0.43 ± 0.01^c^	42.79 ± 0.17^a^	87.64 ± 1.27^e^
SBR0.3-5	51.03 ± 0.15^b^	39.03 ± 0.06^a^	17.03 ± 0.15^d^	3.51 ± 0.18^d^	0.41 ± 0.00^d^	42.59 ± 0.12^a^	91.37 ± 0.33^d^
SBR0.5-3	49.80 ± 0.20^cd^	38.40 ± 0.40^bc^	17.90 ± 0.30^c^	4.67 ± 0.20^b^	0.44 ± 0.01^c^	42.37 ± 0.24^a^	95.70 ± 0.45^c^
SBR0.5-5	50.30 ± 0.20^c^	38.67 ± 0.15^ab^	17.87 ± 0.15^c^	4.14 ± 0.21^c^	0.43 ± 0.01^c^	42.60 ± 0.08^a^	94.90 ± 0.70^c^
SBR0.5-7	49.37 ± 0.35^d^	38.37 ± 0.25^c^	19.27 ± 0.15^a^	5.31 ± 0.44^a^	0.47 ± 0.01^a^	42.70 ± 0.33^a^	100.68 ± 0.82^a^
SBR0.7-5	49.87 ± 0.21^cd^	38.57 ± 0.41^bc^	18.87 ± 0.12^b^	4.70 ± 0.19^b^	0.46 ± 0.01^b^	42.75 ± 0.23^a^	98.60 ± 0.19^b^

NBR, native brown rice powder; SBR, steam exploded brown rice powder; SBR0.3-5, SBR at 0.3 MPa for 5 min; SBR0.5-3, SBR at 0.5 MPa for 3 min; SBR0.5-5, SBR at 0.5 MPa for 5 min; SBR0.5-7, SBR at 0.5 MPa for 7 min; SBR0.7-5, SBR at 0.7 MPa for 5 min. Different letters indicated significant differences at p < 0.05 in the same column. References: Changes in the nutritional value, flavor, and antioxidant activity of brown glutinous rice during fermentation.

### Chemical compounds of brown rice powder

Arabinoxylans, water-extractable arabinoxylans, and reducing sugar contents of different samples are given in Table 2. Arabinoxylans and water-extractable arabinoxylans contents of native brown rice powder were 7.61 and 0.52%, respectively, while that of steam exploded powders were 6.55–7.27% and 0.52–0.55%, respectively. Dietary fiber, mainly included arabinoxylans, is the major constituent in the cell wall of rice bran. The water-extractable arabinoxylans and reducing sugar contents were determined to monitor the effect of the treatments on cell-wall degradation (38). The contents of reducing sugar and water-extractable arabinoxylans in SBR0.5-3 and SBR0.5-7 significantly increased (*p* < 0.05) compared with that of NBR. When the steam explosion intensity was >0.5 MPa for 5 min, it could promote the conversion of insoluble dietary fiber to soluble dietary fiber, which indicated steam explosion had the effect of breaking the cell wall of rice bran (39). Rice bran was characterized by thick walls in which feruloylated arabinoxylans were the predominant polysaccharide components. The degree of arabinoxylans cross-linking is known to be a factor controlling the toughness of plant cell walls (40). Steam explosion could promote the conversion of arabinoxylans to water-extractable arabinoxylans; it broke the crystalline structure of dietary fiber. The increased reducing sugar content may be attributed to the substantial breakdown of starch and the lignocellulosic structure with the removal of cellulose and hemicellulose (41). The lower starch digestibility/bioaccessibility associated with intact cells or tissues (5). As a result, steam explosion might improve the starch digestibility/bioaccessibility of brown rice.

**Table 2:** Chemical composition of native and steam-exploded brown rice powder.

**Samples**	**AX (%)**	**WEAX (%)**	**ReS (mg/g)**	**Protein (%)**	**Starch (%)**	**IBV**	**RDS (%)**	**SDS (%)**	**RS (%)**	**GD (%)**	**Moisture (%)**
NBR	7.61 ± 0.26^a^	0.52 ± 0.01^b^	3.09 ± 0.15^c^	8.29 ± 0.34^ab^	79.36 ± 0.39^a^	10.04 ± 0.38^c^	23.34 ± 0.06^f^	9.01 ± 0.02^a^	47.02 ± 0.45^a^	1.89 ± 0.06^d^	8.18 ± 0.30^ab^
SBR0.3-5	6.89 ± 0.11^bc^	0.52 ± 0.00^b^	3.14 ± 0.09^c^	8.27 ± 0.23^ab^	62.13 ± 0.51^d^	9.19 ± 0.27^d^	36.23 ± 0.01^d^	4.09 ± 0.05^b^	21.81 ± 0.54^d^	8.20 ± 0.16^a^	9.21 ± 1.22^a^
SBR0.5-3	6.89 ± 0.40^bc^	0.52 ± 0.01^b^	3.49 ± 0.08^ab^	7.29 ± 0.65^c^	54.02 ± 0.70^e^	9.81 ± 0.21^c^	34.95 ± 0.05^e^	3.02 ± 0.03^d^	16.05 ± 0.68^e^	2.40 ± 0.04^c^	6.89 ± 0.39^b^
SBR0.5-5	6.78 ± 0.20^bc^	0.55 ± 0.03^a^	3.29 ± 0.09^bc^	7.80 ± 0.05^bc^	69.67 ± 1.08^c^	9.95 ± 0.14^c^	38.89 ± 0.03^c^	3.83 ± 0.05^c^	26.95 ± 1.12^c^	6.68 ± 0.14^b^	7.05 ± 0.99^b^
SBR0.5-7	7.27 ± 0.29^ab^	0.55 ± 0.01^a^	3.74 ± 0.31^a^	8.39 ± 0.21^ab^	77.78 ± 0.51^ab^	13.09 ± 0.14^a^	41.56 ± 0.10^a^	1.70 ± 0.03^f^	34.52 ± 0.43^b^	8.54 ± 0.36^a^	6.53 ± 1.16^b^
SBR0.7-5	6.55 ± 0.20^c^	0.55 ± 0.01^a^	3.34 ± 0.08^bc^	8.45 ± 0.16^a^	77.33 ± 1.59^b^	11.85 ± 0.17^b^	40.39 ± 0.09^b^	2.62 ± 0.08^e^	34.33 ± 1.61^b^	1.98 ± 0.01^d^	6.50 ± 0.37^b^

NBR, native brown rice powder; SBR, steam exploded brown rice powder; SBR0.3-5, SBR at 0.3 MPa for 5 min; SBR0.5-3, SBR at 0.5 MPa for 3 min; SBR0.5-5, SBR at 0.5 MPa for 5 min; SBR0.5-7, SBR at 0.5 MPa for 7 min; SBR0.7-5, SBR at 0.7 MPa for 5 min; AX, arabinoxylans; WEAX, water-extractable arabinoxylans; ReS, Reducing sugar; IBV, Iodine blue value; RDS, rapidly digestible starch; SDS, slowly digestible starch; RS, resistant starch; GD, gelatinization degree. Different letters indicated significant differences at p < 0.05 in the same column.

The iodine blue value can reflect the degree of cell damage, the larger iodine blue value indicated the more free starch and the higher degree of cell damage. The brown rice powder showed a larger amylose content dissolved in solution and the more starches gelatinized during the cooking process, indicated the better eating quality of the rice (3, 42). Steam explosion significantly increased the iodine blue value of brown rice powder at 0.5 MPa for 7 min and 0.7 MPa for 5 min (*p* < 0.05), which indicated that steam explosion assisted milling damaged cell and starch structure. High-temperature treatment could improve the quality of brown rice, the iodine blue value of high-temperature air fluidization-treated brown rice at 130°C increased significantly, indicating that the quality of brown rice is higher than that of untreated brown rice (3). Gelatinization of starch played an important role in digestion by improving the ability of starch to absorb water and enabling enzymes to degrade starch, thereby increased starch digestibility. Compared with native brown rice powder (1.89%), the degree of gelatinization of steam exploded samples was gradually increased with the rising of treated time, indicated the occurrence of partial disruption of starch granules during treatment with saturated steam. The degree of gelatinization increased from 2.40 to 8.53% with the increase of treated time from 3 to 7 min at 0.5 MPa. The degree of gelatinization of steam exploded powders was gradually decreased from 8.20 to 1.97% with the rising of treated pressure from 0.3 to 0.7 MPa for 5 min. These results indicated that the starch gelatinized during steam explosion processing might create the gel network structure that can enhance the structural strength (43).

The protein and starch contents of brown rice powders were ranged from 7.29 to 8.45% and 54.02 to 79.36%, respectively. Steam explosion significantly increased (*p* < 0.05) the rapidly digestible starch content, however, significantly decreased (*p* < 0.05) the slowly digestible starch and resistant starch content of brown rice powders. The rapidly digestible starch and resistant starch of steam exploded powders were gradually increased from 36.23 to 40.39% and 21.81 to 34.33% with the rising of treated pressure from 0.3 to 0.7 MPa for 5 min. The rapidly digestible starch and resistant starch of steam exploded powders were gradually increased from 34.95 to 41.56% and 16.05 to 34.52% with the rising of treated time from 3 min to 7 min at 0.5 MPa. The slowly digestible starch content of steam exploded samples was gradually decreased from 4.09 to 1.07% with the rising of treated pressure from 0.3 to 0.7 MPa for 5 min. The rapidly digestible starch content increased and slowly digestible starch and resistant starch content of brown rice powder decreased after steam explosion treatment, which could be due to steam explosion and broken the complex physicochemical mechanisms, which was known as “biomass recalcitrance” for protecting its structural carbohydrates from attack by enzymes (7), resulting in a higher rapidly digestible starch content. The steam exploded brown rice powder (0.5 MPa, 7 min) had the higher content of water-extractable arabinoxylans, reducing sugar, rapidly digestible starch, and degree of gelatinization.

### *In vitro* starch digestibility of brown rice powder

**Figure 3:**
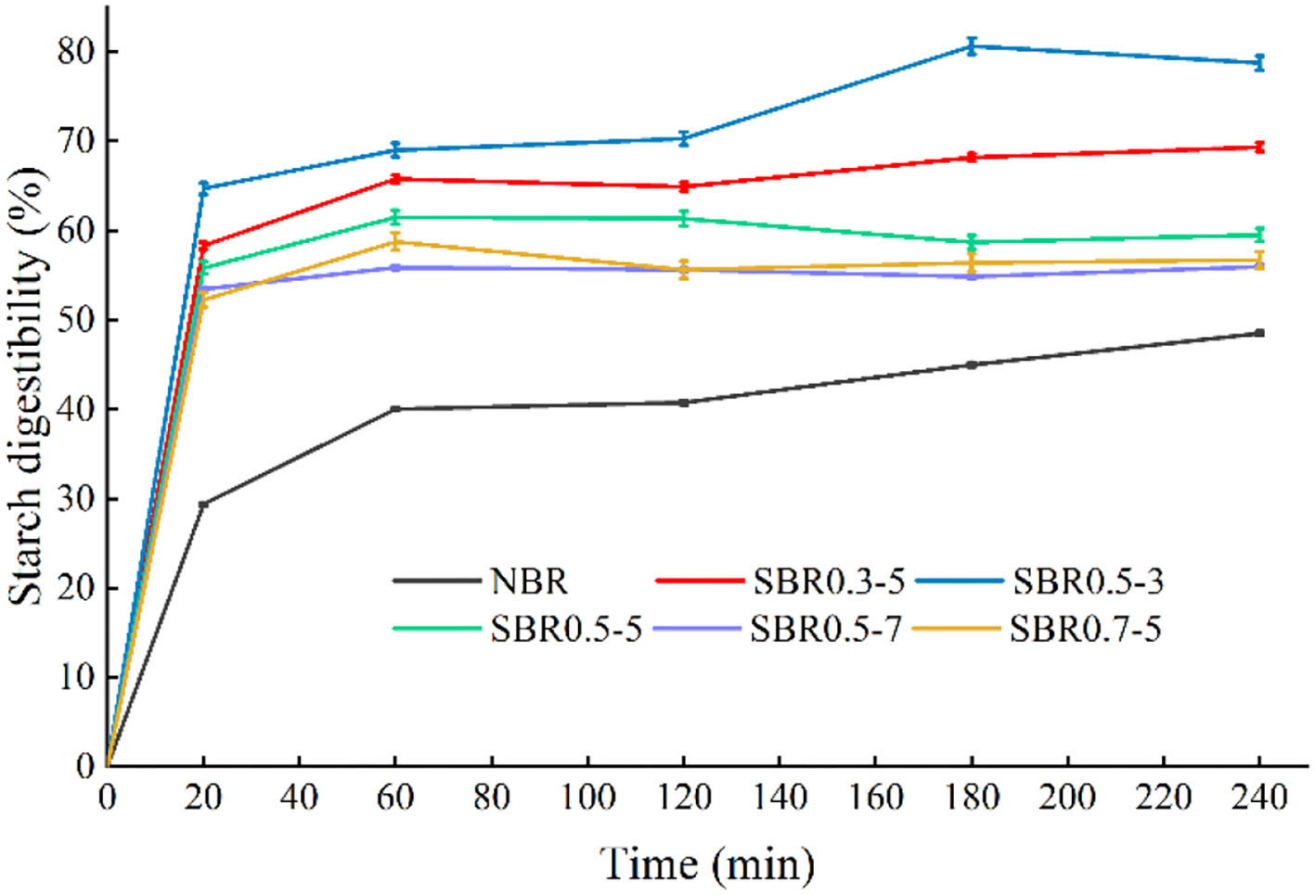
Effect of steam explosion on *in vitro* starch digestibility of brown rice powder. NBR, native brown rice powder; SBR, steam exploded brown rice powder; SBR0.3-5, SBR at 0.3 MPa for 5 min; SBR0.5-3, SBR at 0.5 MPa for 3 min; SBR0.5-5, SBR at 0.5 MPa for 5 min; SBR0.5-7, SBR at 0.5 MPa for 7 min; SBR0.7-5, SBR at 0.7 MPa for 5 min.

Brown rice is rich in nutrition and has the potential to be used as raw material for food production for infants and the elderly. However, due to the existence of rice bran layer, the indigestible characteristics of rough rice food limit the expansion of its target consumer groups. Starch was one of the main nutrients in brown rice powder, and its digestibility directly influenced the digestion and absorption of powder (6). In the *in vitro* digestion process, the enzymatic hydrolysis rate of all brown rice powders increased rapidly in the first 20 min, and stabilized after 60 min, which was in line with human digestible characteristics (31). After saturated steam high pressure and high temperature of steam explosion treatment, brown rice powder formed a more unstable structure. The highest hydrolysis rate of brown rice starch was between 0 and 20 min. In this period, the highest hydrolysis rate of starches was found for steam exploded brown rice powder at 0.5 MPa for 3 min (3.24% starch/min), followed by 0.3 MPa for 5 min (2.92% starch/min), 0.5 MPa for 5 min (2.79% starch/min), and finally at 0.5 MPa for 7 min and 0.7 MPa for 5 min (2.61–2.67% starch/min) (Figure 3). From 20 to 60 min, there was a remarkable drop in the hydrolysis rate particularly for steam exploded brown rice powder at 0.5 MPa for 7 min (0.06% starch/min), whereas native powder in this period had the highest value (0.27% starch/min) and with other samples in between (0.11 and 0.19% starch/min). The lowest ultimate starch digestibility (55.98%) of steam exploded brown rice powder was obtained for the condition of steam explosion at 0.5 MPa for 7 min. In the previous study, steam explosion significantly changed physical structure of solid medium, which possessed a more porous structure, provided more effective contact area for enzymes and microorganisms, thus improved the enzymatic hydrolysis and fermentation performance (44, 45).

### Solvent retention capacity of brown rice powder

**Figure 4:**
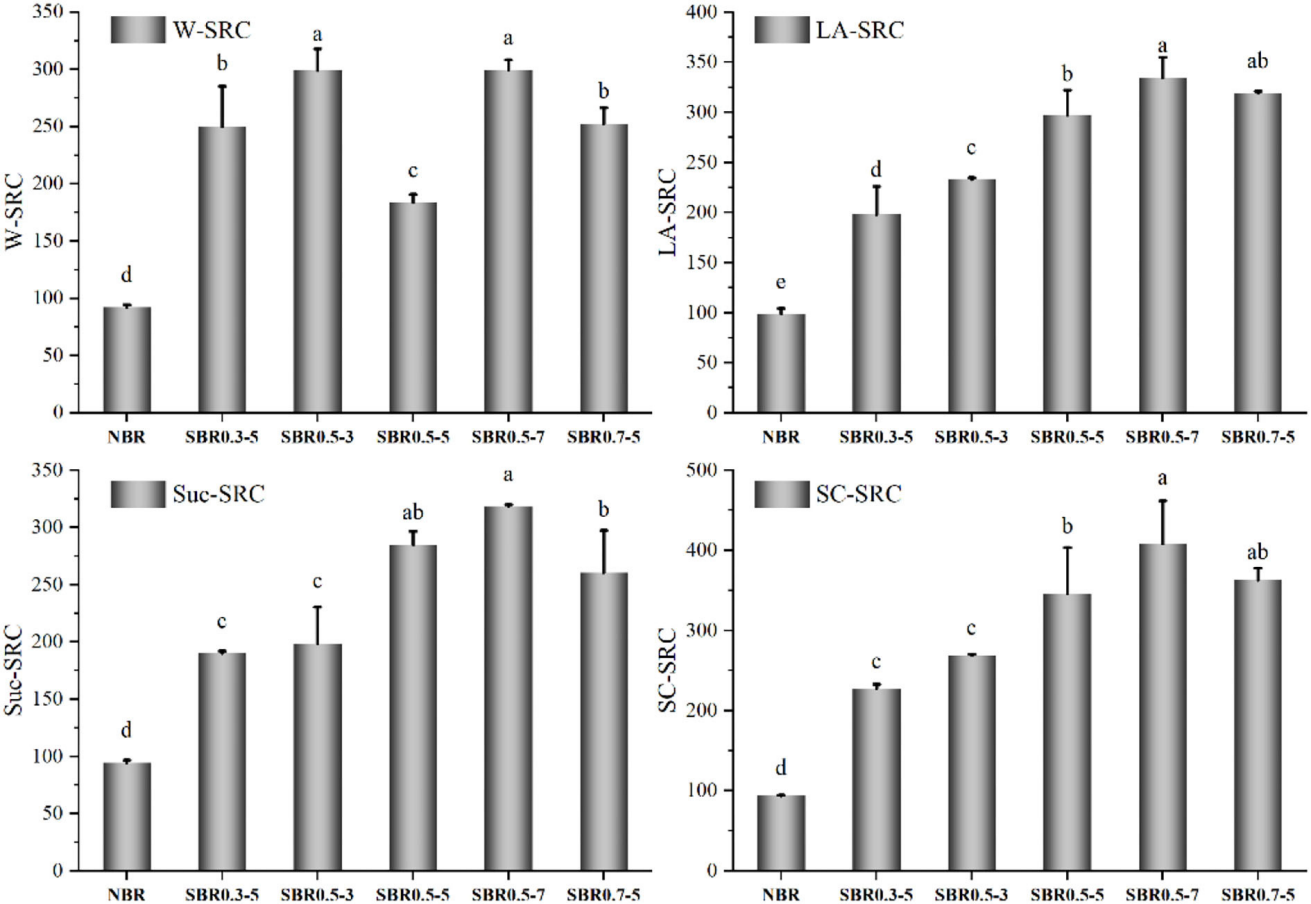
Effect of steam explosion on the solvent retention capacity (SRC) values of brown rice powder. Suc-SRC, sucrose SRC; SC-SRC, sodium carbonate SRC; LA-SRC, lactic acid SRC; W-SRC, water SRC; NBR, native brown rice powder; SBR, steam exploded brown rice powder; SBR0.3-5, SBR at 0.3 MPa for 5 min; SBR0.5-3, SBR at 0.5 MPa for 3 min; SBR0.5-5, SBR at 0.5 MPa for 5 min; SBR0.5-7, SBR at 0.5 MPa for 7 min; SBR0.7-5, SBR at 0.7 MPa for 5 min (Means that do not share a letter are significantly different, *p* < 0.05).

SRC was deemed to be an efficient method to evaluate wheat flour quality; it is a special method for predicting the flour function by estimating the relative contributions of individual flour compounds to water absorption (46), which also used to evaluation of oat flour properties (47). SRC values of brown rice powders are summarized in Figure 4. LA-SRC, Suc-SRC, and SC-SRC changed significantly as the treatment time was extended from 3 to 7 min. The content of extractable arabinoxylans and damaged starch content increased by steam explosion treatment based on Suc-SRC, SC-SRC, and W-SRC. Steam explosion destroyed the crystalline structure of brown rice powder, allowed more hydroxyl groups bound with water and led to an increase in SRC. Since water retention capacity of brown rice powder was significantly enhanced by steam explosion, the brown rice powder would be more fully wetted than that of native, which might be attributed to solve the agglomeration problem of cereal flour. Similar trends have been reported for steam exploded blue-grained whole meal flour (13). There was a remarkable negative correlation (*p* < 0.05) between SDS and W-SRC (r = −0.916), LA-SRC (r = −0.904), Suc-SRC (r = −0.870), SC-SRC (r = −0.916), while water-extractable arabinoxylans and LA-SRC (r = 0.860, *p* < 0.05), Suc-SRC (r = 0.862, *p* < 0.05), SC-SRC (r = 0.843, *p* < 0.05), RDS and LA-SRC (r = 0.954, *p* < 0.01), Suc-SRC (r = 0.942, *p* < 0.01), SC-SRC (r = 0.958, *p* < 0.01) showed significant positive relationship. Brown rice powder modification by steam explosion at 0.5 MPa for 7 min had the highest W-SRC (299.42%), LA-SRC (334.55%), Suc-SRC (318.33%), and SC-SRC (408.42%), respectively, that of native were 92.30, 98.60, 94.51, and 93.56%, respectively.

### Textural property of brown rice paste

**Figure 5:**
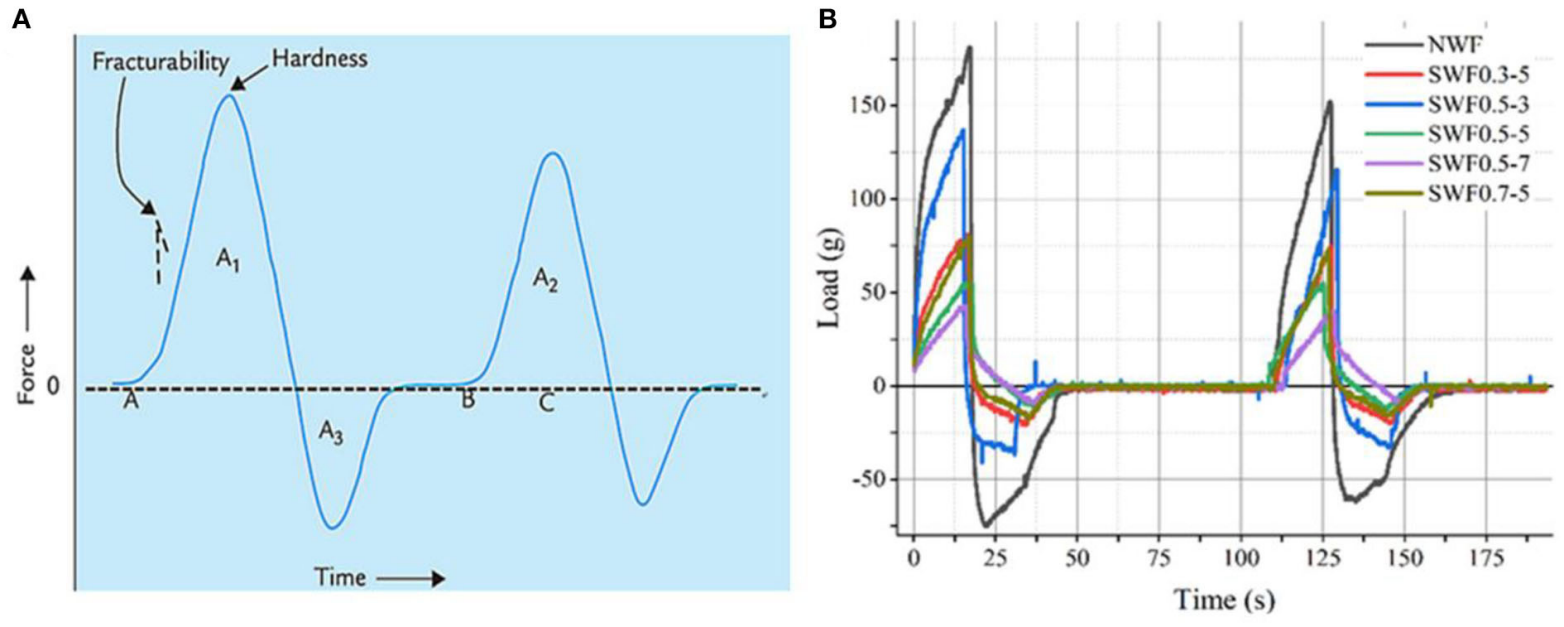
A typical texturometer curve **(A)** (cohesiveness = A_2_/A_1_, adhesiveness = A_3_) (49) and loading image of brown rice pastes **(B)**. NBR, native brown rice powder; SBR, steam exploded brown rice powder; SBR0.3-5, SBR at 0.3 MPa for 5 min, SBR0.5-3, SBR at 0.5 MPa for 3 min; SBR0.5-5, SBR at 0.5 MPa for 5 min; SBR0.5-7, SBR at 0.5 MPa for 7 min; SBR0.7-5, SBR at 0.7 MPa for 5 min.

Textural property was a general term for non-taste sensorial property, and it was directly related to physicochemical property of foods, which directly affected the mouth-feel, flavor release, and sensorial pleasure of foods and influenced consumer preferences (48). The paste texture properties of brown rice powder are shown in Figure 5. The hardness of paste decreased with steam explosion. Compared to native powder, the hardness of steam exploded pastes decreased from 181 to 43–137 g. These results reported that steam exploded brown rice powder had a higher gelation concentration than native powder. Powder derived from steam exploded brown rice would be useful in food systems such as pastes that require thinning. Additionally, fracturability, gumminess, chewiness, and cohesiveness had the same trend as hardness (Pearson correlation, r = 0.993, 0.997, 0.987, and 0.921, respectively, *p* < 0.01). Gumminess recognized as the energy is required to destroy a semi-solid food to a state ready for swallowing. With semisolid food products, hardness was low (49). The textural structure of paste was unstable and with weakened mechanical strength. Results showed that the anticipated oral sensory of rice paste was obtained at 0.5 MPa for 7 min, while the corresponding hardness, fracturability, gumminess, chewiness, cohesiveness, and adhesiveness of the rice paste were 43.00 g, 10.00 g, 35.00 g, 2.50 mJ, 0.82, and 0.40 mJ, respectively.

### Dynamic rheological property of brown rice paste

**Figure 6:**
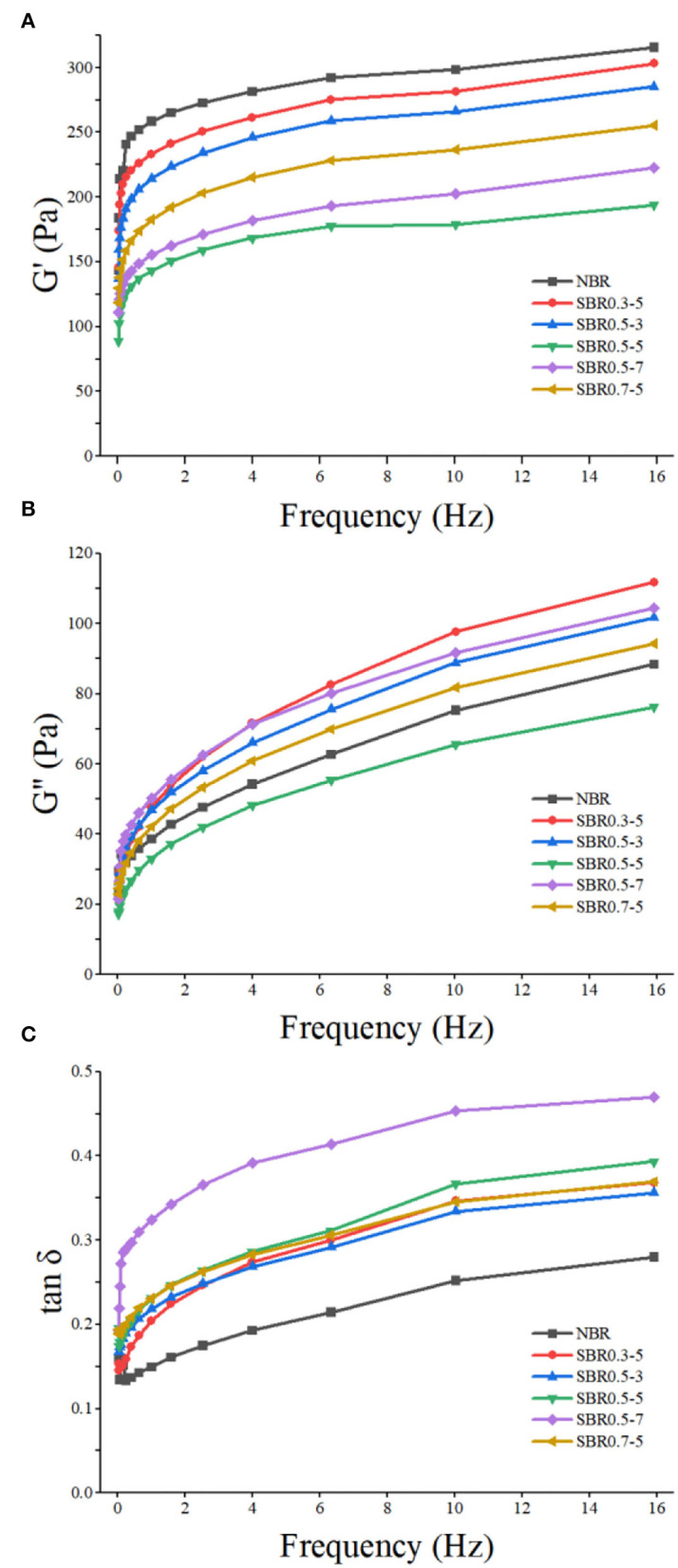
Rheograms of dynamic rheological properties of brown rice paste during frequency sweep. **(A)** G' of samples, **(B)** G” of samples, **(C)** tan δ of samples. NBR, native brown rice powder; SBR, steam exploded brown rice powder; SBR0.3-5, SBR at 0.3 MPa for 5 min; SBR0.5-3, SBR at 0.5 MPa for 3 min; SBR0.5-5, SBR at 0.5 MPa for 5 min; SBR0.5-7, SBR at 0.5 MPa for 7 min, SBR0.7-5, SBR at 0.7 MPa for 5 min.

Rheological test results were derived from the combined and cumulative contributions of the flour functional compounds rather than assessed the effect of mixture function by individual flour polymers (50). Viscoelasticity was one of the important eating qualities of brown rice paste. Figure 6 shows the storage modulus (G'), loss modulus (G”), and loss factor (tanδ) of native and steam exploded brown rice paste. Both storage modulus and loss modulus of native and steam exploded brown rice paste increased with frequency increase; the elasticity and viscosity were frequency-dependent (33). Compared with native brown rice paste, the storage modulus and loss modulus of steam exploded paste (0.5 MPa, 5 min) were lower throughout the frequency; steam explosion (0.5 MPa, 5 min) reduced elasticity and viscosity of brown rice paste, which weaken the strength of it (33). On the contrary, the storage modulus and loss modulus of the steam exploded paste (0.3 MPa, 5 min) were higher than those of the native paste. Steam explosion could increase the viscosity-to-elasticity ratio based on the results of loss factor; this indicated that the steam exploded paste has low elasticity than the native (48). But the inherent rheological property was not changed, with the same trend of loss factor <1 (28). The viscoelastic properties of steam exploded paste (0.5 MPa, 3 min) were the closest to the native, other conditions of steam explosion treatment was conductive to modifying the natural viscoelastic properties of brown rice paste, and the lowest loss factor of steam exploded paste was at 0.5 MPa for 7 min. The results indicated that the fluid properties of steam exploded powder are enhanced, which were compatible with the textural property analysis above.

## Conclusion

The nutritional component, physicochemical, and rheological properties of brown rice powder were altered by the thermal-mechanical action of steam explosion. Steam explosion destroyed the cell wall by facilitating the conversion of water-unextractable to water-extractable arabinoxylans and increased the reducing sugar content. Therefore, the brown rice powder treated by steam explosion was more accessible for digestible process. Steam explosion at 0.5 MPa for 7 min showed the highest water-extractable arabinoxylans and reducing sugar content, which might be conducive to the highest rapidly digestible starch content. The thermal and mechanical effect produced through steam explosion led to a change in the order of brown rice starch, which resulted in a change in crystals type, and the degree of gelatinization, thereby improving the rheological characteristics of pastes. Results showed that steam explosion was beneficial in improving SRC, iodine blue value, and the degree of gelatinization at 0.5 MPa for 7 min. And the paste possessed the anticipated oral sensory and lowest loss factor. Steam explosion processing will offer a new approach for cereal powder preparation, and steam exploded brown rice powder (0.5 MPa, 7 min) could be widely used in food products.

## Data availability statement

The original contributions presented in the study are included in the article/supplementary material, further inquiries can be directed to the corresponding authors.

## Author contributions

FK and XG: conceptualization, resources, and supervision. FK, YL, and QZ: methodology and validation. FK: software, formal analysis, data curation, writing-original draft preparation, writing-review and editing, project administration, and funding acquisition. QZ, FK, XD, YD, and YL: investigation. QZ and FK: visualization. All authors have read and agreed to the published version of the manuscript.

## Funding

This study was financially supported by the Open Project of Liaocheng University Animal Husbandry Discipline (No. 319312101-08), the Doctoral Research Startup Foundation of Liaocheng University (No. 318052122), the Research and Development of Key Technology of Nutrition Enhancement of Bone-derived Pet Food (No. K22LD04), and the Project of Shandong Province Higher Educational Science and Technology Program for Youth (No. 2019KJF028).

## Conflict of interest

The authors declare that the research was conducted in the absence of any commercial or financial relationships that could be construed as a potential conflict of interest.

## Publisher's note

All claims expressed in this article are solely those of the authors and do not necessarily represent those of their affiliated organizations, or those of the publisher, the editors and the reviewers. Any product that may be evaluated in this article, or claim that may be made by its manufacturer, is not guaranteed or endorsed by the publisher.
